# Using deep learning artificial intelligence for sex identification and taxonomy of sand fly species

**DOI:** 10.1371/journal.pone.0320224

**Published:** 2025-04-03

**Authors:** Mohammad Fraiwan, Rami Mukbel, Dania Kanaan

**Affiliations:** 1 Department of Computer Engineering, Jordan University of Science and Technology, Irbid, Jordan; 2 College of Veterinary Medicine, Jordan University of Science and Technology, Irbid, Jordan; World Health Organization, Regional Office for South-EastAsia, INDIA

## Abstract

Sandflies are vectors for several tropical diseases such as leishmaniasis, bartonellosis, and sandfly fever. Moreover, sandflies exhibit species-specificity in transmitting particular pathogen species, with females being responsible for disease transmission. Thus, effective classification of sandfly species and the corresponding sex identification are important for disease surveillance and control, managing breeding/populations, research and development, and conducting epidemiological studies. This is typically performed manually by observing internal morphological features, which maybe an error-prone tedious process. In this work, we developed a deep learning artificial intelligence system to determine the gender and to differentiate between three species of two sandfly subgenera (i.e., *Phlebotomus alexandri*, *Phlebotomus papatasi*, and *Phlebotomus sergenti*). Using locally field-caught and prepared samples over a period of two years, and based on convolutional neural networks, transfer learning, and early fusion of genital and pharynx images, we achieved exceptional classification accuracy (greater than 95%) across multiple performance metrics and using a wide range of pre-trained convolutional neural network models. This study not only contributes to the field of medical entomology by providing an automated and accurate solution for sandfly gender identification and taxonomy, but also establishes a framework for leveraging deep learning techniques in similar vector-borne disease research and control efforts.

## Introduction

A sandfly is a tiny insect belonging to the family Psychodidae. They are found in sandy areas, particularly in tropical and subtropical regions, and are known for their painful bites. Sandflies are active mainly during dusk and dawn hours and are most commonly found in rural or wilderness areas. They are the only psychodid insects that possess piercing mouthparts, which enable them to feed on blood. These insects are small, usually measuring 1–3 mm in length, and are difficult to see with the naked eye. Some species of sandflies are also known to transmit diseases such as leishmaniasis and bartonellosis to humans and animals [1]. Identifying sandflies to the species level is challenging and often requires the examination of their internal structures [2,3]. The species of sand flies are classified into 23 genera, of which six are the most significant. These include *Phlebotomus*, *Sergentomyia*, and *Chinius* in the Old World, and *Lutzomyia*, *Brumptomyia*, and *Warileya* in the New World. Old World refers to Afrotropical, Palaearctic, Malagasy, Oriental and Australian regions. The New World refers to Nearctic and Neotropical regions [4,5]. There are many different species of sandflies, and they can vary depending on the geographic location. Some of the most common species of sandflies are:

- *Lutzomyia longipalpis*: This species is found in Central and South America and is the main vector for the parasite that causes visceral and cutaneous leishmaniasis.
- *Phlebotomus papatasi*: This species is found in the Middle East, Central Asia, and North Africa, and is the primary vector for the parasite that causes cutaneous leishmaniasis.
- *Phlebotomus argentipes*: This species is found in India, Nepal, and Bangladesh and is the main vector for the parasite that causes visceral leishmaniasis.
- *Sergentomyia*: This genus is found throughout the world.

Sandflies are known to transmit a variety of diseases that can cause fever, headaches, muscle pain, anemia, skin lesions, and skin rash [2], including:

- Leishmaniasis: This parasitic disease is caused by *Leishmania* and transmitted to humans and animals through the bites of infected sandflies.
- Bartonellosis: This bacterial infection, caused by *Bartonella bacilliformis*.
- Pappataci and sandfly fever: This viral disease, caused by the phlebovirus.

Sandflies have a specific relationship with the pathogens they transmit. Each species of sandfly is associated with the transmission of particular species of pathogens, meaning that a given sandfly species will only transmit certain pathogen species, not others. This specificity implies a close match between the sandfly species and the pathogens they carry. For example, *Ph. alexandri* transmit *Leishmania donovani*, whereas *Ph. papatasi* transmit *Leishmania major*. Furthermore, only female sandflies are capable of biting humans and transmitting diseases. Female sandflies require a blood meal to develop their eggs, so they are the ones that actively seek out hosts to feed on. Male sandflies typically feed on plant nectar and are not involved in disease transmission. Hence, determining the sex of sandflies is important for understanding disease transmission dynamics and risks, analyzing the efficacy of control measures, managing breeding/population, and conducting epidemiological studies. This typically requires close examination of the sandfly physical characteristics, which can be challenging and may require a trained entomologist or compound microscope to accurately identify the differences. Although male and female sandflies have some differences in their internal physical appearance, these distinctions can be subtle and may require practice to identify accurately. Nonetheless, there are some general characteristics that can help determine the sex of sandflies, including:

1. Body size: Female sandflies are typically larger than males, although the size difference can be small and may not be noticeable without close inspection.
2. Abdomen shape: In female sandflies, the abdomen is usually more rounded and protruding, while in males, it is more elongated and pointed.
3. Genitalia: The genitalia of male and female sandflies are located at the rear of the abdomen and are different in shape and structure. In males, the genitalia consist of a pair of claspers, which are used to hold onto the female during mating. In females, the genitalia are more complex and include the ovipositor, called the spermatheca, which is used to lay eggs.
4. Antennae: In some species of sandflies, the antennae of males are larger and more conspicuous than those of females.

To the best of our knowledge, there have been hardly any studies on using artificial intelligence (AI) to determine the sex and species of sandflies, with typical studies relying on spectrometry systems to achieve this task [6,7]. However, there have been some studies using computerized methods to identify and classify sandfly species based on morphological characteristics, which could potentially be extended to include sex determination. In this context, Hakan Kavur [8] proposed an online system that allows users to enter 21 qualitative and quantitative parameters related to morphological features, which are then used to identify the species and sex of the sandfly using a programmed identification key (i.e., flowchart). Over the past decade, deep learning algorithms have gained significant traction, revolutionizing applications such as medical imaging [9], veterinary medicine [10], agriculture [11], autonomous driving, and natural language processing. Their ability to learn complex patterns from data has transformed traditional methods into efficient and automated systems. Such systems are continuously improving and gaining wider public acceptance as they have the potential to reduce errors and save effort [12]. In the context of this work, a recent study used a deep learning algorithm to analyze images of sandfly specimens and employed computer vision techniques to classify sandfly species based on features such as wing shape and color [13]. However, the reported accuracy was around 77%, which is considered low for an AI system. While these studies did not specifically address sex determination, they demonstrate the potential of AI and machine learning in automating the identification and classification of sandflies.

**Fig 1:**
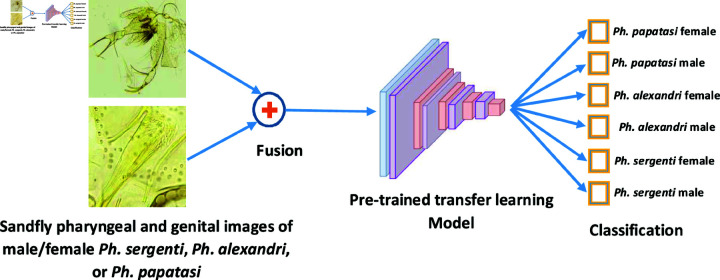
An illustration of a typical usage scenario of the proposed system.

**Fig 2:**
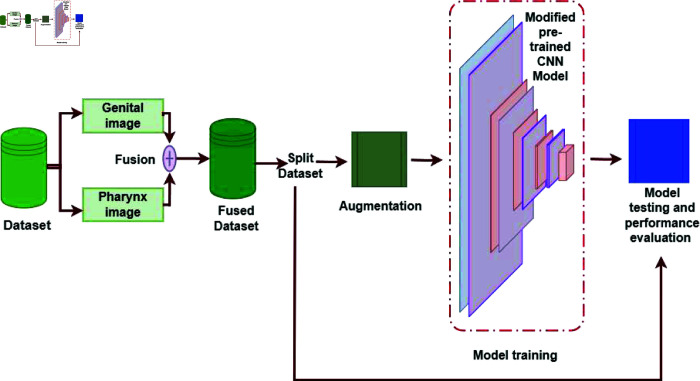
The steps undertaken to develop the sandfly sex identification and taxonomy model.

This research is a culmination of two years’ effort to locally gather, process, identify, and photograph relevant features of the most dominant sandfly species in Jordan. The goal was to develop highly accurate image-based AI systems for the sex identification and taxonomy of sandfly species. This work advances the field by addressing key limitations and leveraging recent AI advancements in species identification, while also introducing novel techniques that set it apart from existing literature. Traditional methods for identifying sandfly species and gender are manual, relying on observation of internal morphological features, which is time-consuming and error-prone; the AI-driven approach used in this work automates this process, reducing human error and dependence on expert input. Building upon recent developments in deep learning, this study uses convolutional neural networks (CNNs) and transfer learning to achieve high classification accuracy, reaching over 95% across multiple performance metrics. This level of precision exceeds or matches current standards in the field [14]. Additionally, a unique aspect of this approach is the early fusion of genital and pharynx images, a method that captures subtle morphological distinctions between sandfly species and genders more effectively than single-image models, and which could be applied to other vector species. Fig 1 shows a typical usage scenario for the proposed system. Ultimately, this research aligns closely with practical applications such as disease surveillance, vector control, and epidemiological studies, positioning it not just as a technical contribution but as a tool with tangible public health benefits, especially in managing vector-borne diseases (e.g., leishmaniasis). Fig 2 shows a general diagram of the steps used to develop and test the convolutional neural network models. In the next few sections, we detail each element in the process. The materials and methods section provides more information about the dataset, the CNN pre-trained models, and the performance evaluation metrics and setup. The results are presented and discussed in the results section. We conclude in the last section.

## Materials and methods

### Ethical approval

This study was approved by the research committee at the Faculty of Veterinary Medicine and Deanship of Academic Research, Jordan University of Science and Technology, under the research proposal No. 244/2020. According to Jordanian regulations, no special approval or permit is required to collect insects in Jordan.

### Dataset

**Fig 3:**
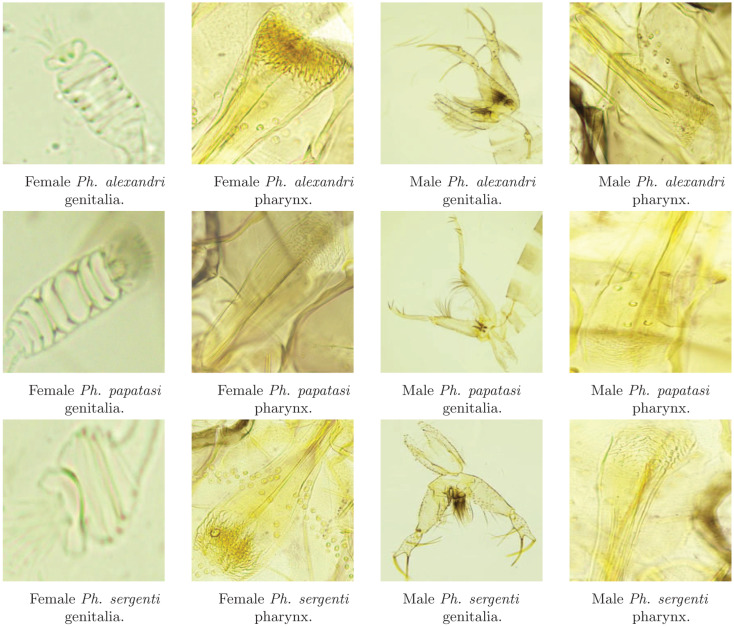
Sample images of the three sand fly species for both males and females.

The sandflies were collected over a period of two years from various regions in northern Jordan, resulting in a total of 758 sandfly specimens. Two pictures were taken per specimen: one of the pharynx and the other of the genital part. The number of specimens per species per sex was as follows: 106 female *Ph. alexandri*, 85 male *Ph. alexandri*, 269 female *Ph. papatasi*, 158 male *Ph. papatasi*, 45 female *Ph. sergenti*, and 95 male *Ph. sergenti*. The sex and species were determined by two experts in the field. Some rarer species were captured but discarded after identification due to their low numbers, as they would be useless for AI model development. Furthermore, many pictures were discarded because the 2D microscope was not able to capture the features of interest in the 3D slide. This resulted in 758 pharynx images and 758 genital images, see Fig 3. The detailed process of collecting the sandflies, processing the specimens, photographing the body parts, and classifying the images is part of a separate data article and is beyond the scope of this work.

### Convolutional neural network models

In the past decade, a multitude of CNN architectures have been developed, each offering unique advantages in terms of performance, efficiency, and scalability. In this section, we provide a comprehensive analysis of several prominent CNN architectures, including Darknet, GoogLeNet, Inception, MobileNet, NASNet, ShuﬄeNet, SqueezeNet, and Xception. By design, these models were trained by their respective creators to classify 1000 possible objects (e.g., screwdriver versus cat) based on the ImageNet dataset [15]. This dataset is completely different from the application in this work, and the types of objects sought here (i.e., sandfly species) are not included in the dataset. However, the design of these CNN architectures captures generic features in the initial layers (e.g., colors, edges, etc.) and specific details in later layers that are closer to the output. Hence, the power of transfer learning is to utilize the learned generic knowledge and target the model retraining toward specific applications. To this end, the size of the input layer of each CNN architecture was modified to the fused image size (i.e., 400 × 800 × 3). Furthermore, the learnable layer before the output layer was replaced by either a fully connected layer or a 2D convolutional layer, depending on the specific CNN architecture. Additionally, a new classification layer with 6 output categories (i.e., male/female for the three species) replaces the existing classification layer in each model, which was originally tailored for the aforementioned 1000 objects. We examine the architectural features, design principles, and computational characteristics of each model, highlighting their respective strengths and weaknesses. By comparing these architectures across various dimensions such as parameter efficiency, computational cost, and accuracy, we aim to provide insights into the diverse landscape of deep learning models and their suitability for different applications and deployment scenarios.

Darknet-19 [16] is a high-performance CNN architecture specifically developed for real-time object detection in the YOLO (You Only Look Once) system. It consists of 19 convolutional layers and 5 max-pooling layers, incorporating 3 × 3 convolutional filters to capture fine-grained spatial features and 1 × 1 filters to reduce dimensionality and enhance computational efficiency. Each convolutional layer is followed by batch normalization, which mitigates internal covariate shift and accelerates convergence during training. This feeds the leaky ReLU activation functions, which address the dying ReLU problem by allowing a small gradient when the unit is not active. Darknet-19 uses global average pooling instead of fully connected layers, reducing the number of parameters and overfitting risk.

Darknet-53’s [16] design makes it significantly more powerful than its predecessor, Darknet-19, in terms of both accuracy and efficiency. It balances the need for a deep network to capture complex patterns with optimizations that ensure real-time performance. Key features include: (1) Residual Connections: Introduced to address the vanishing gradient problem and enable the training of deeper networks by allowing gradients to flow through shortcut connections. (2) Convolutional Layers: Consists exclusively of 3 × 3 and 1 × 1 convolutional filters, optimized to capture fine-grained details and reduce computational complexity. (3) Batch Normalization: Applied after every convolutional layer to stabilize and speed up the training process. (4) Leaky ReLU Activation: Used throughout the network to introduce non-linearity and improve learning dynamics by allowing a small gradient when the unit is inactive. (5) Feature Pyramid Network (FPN): Utilized to detect objects at multiple scales, enhancing the network’s ability to handle objects of varying sizes. (6) Global average pooling.

DenseNet (Densely Connected Convolutional Networks) [17] is a CNN architecture designed to maximize information flow and gradient propagation through the network. It consists of dense blocks where each layer is directly connected to every other layer in a feed-forward fashion. This dense connectivity pattern means that the *l^th^* layer receives the feature maps of all preceding layers $\left(x_{0},x_{1},\ldots ,x_{l-1}\right)$ as input, enhancing feature reuse and substantially reducing the number of parameters compared to traditional architectures. Key characteristics include: (1) Dense connectivity: Each layer has direct access to the gradients from the loss function and the original input signal, which alleviates the vanishing gradient problem. (2) Composite function: Each layer comprises batch normalization, followed by a ReLU activation and a 3 × 3 convolution. (3) Bottleneck layers: 1 × 1 convolutions are used to reduce the number of input feature maps before 3 × 3 convolutions, enhancing computational efficiency. (4) Transition layers: These layers, consisting of batch normalization, a 1 × 1 convolution, and a 2 × 2 average pooling layer, are employed between dense blocks to manage complexity and downsample feature maps. (5) Growth rate: The number of output feature maps of each layer, known as the growth rate, is kept relatively small to control the model size.

GoogLeNet [18], also known as Inception v1, is an efficient convolutional neural network designed for image classification and object detection, featuring the innovative Inception module which combines 1 × 1, 3 × 3, and 5 × 5 convolutions along with 3 × 3 max pooling within a single module to capture multi-scale features. It employs 1 × 1 convolutions to reduce dimensionality before expensive convolutions, enhancing computational efficiency and reducing parameters. The model includes auxiliary classifiers to mitigate the vanishing gradient problem, providing extra gradient signals and acting as regularizers. Additionally, global average pooling is used. These design choices enable GoogLeNet to achieve high performance with lower computational cost, influencing many subsequent deep learning architectures.

Inception v3 [19], a significant evolution from its predecessor Inception v1, introduces several architectural advancements that further enhance its performance and efficiency. The key innovations include: (1) Factorized convolutions: Inception v3 utilizes factorized convolutions to reduce computational cost while maintaining expressive power. This involves decomposing large convolutions (e.g., 5 × 5) into smaller convolutions (e.g., 3 × 3) to capture spatial relationships more efficiently. (2) Asymmetric convolutions: Asymmetric convolutions, such as 3 × 3 convolutions decomposed into 1 × 3 and 3 × 1 convolutions, are employed to improve parameter efficiency and increase model capacity. (3) Extensive batch normalization: Batch normalization is applied more extensively throughout the network, stabilizing training and accelerating convergence by normalizing activations within each mini-batch. (4) Auxiliary classifiers and grid size reductions: Inceptionv3 maintains auxiliary classifiers and introduces additional grid size reductions to address the challenges of vanishing gradients and handle feature maps of varying sizes effectively. (5) Efficient Inception modules: The architecture of the Inception modules is further optimized to strike a balance between computational efficiency and representational power, ensuring effective feature extraction across multiple scales.

MobileNetV2 [20] is a CNN network architecture optimized for mobile and embedded devices, offering high performance with low computational cost and memory footprint. It introduces several key innovations, including inverted residual blocks with linear bottlenecks, which allow for efficient information flow and reduced parameter count. Depthwise separable convolutions further enhance efficiency by decoupling spatial and channel-wise convolutions, reducing computational complexity. MobileNetV2 also incorporates linear bottleneck layers and shortcut connections, facilitating gradient flow and improving feature representation. Additionally, it utilizes width multiplier and resolution multiplier techniques to customize the model’s size and computational requirements, making it adaptable to various resource constraints. Overall, MobileNetV2 achieves excellent performance on image classification and other computer vision tasks while being highly efficient, making it ideal for deployment on resource-constrained devices.

NASNetMobile [21], short for Neural Architecture Search Network Mobile, is a cutting-edge convolutional neural network architecture designed through neural architecture search (NAS), aiming to optimize both performance and efficiency for mobile and embedded devices. Key features include a cell-based architecture, where cells are stacked to form the network, and the use of reinforcement learning to guide the search process towards architectures with superior performance. NASNetMobile introduces the concept of learned separable convolutions, which enable the network to learn more efficient representations by decoupling spatial and channel-wise convolutions. The architecture also incorporates various architectural innovations, such as skip connections and densely connected blocks, to enhance feature reuse and gradient flow, leading to improved accuracy and robustness. By leveraging these advancements, NASNetMobile achieves state-of-the-art performance on image classification tasks while maintaining low computational cost and memory footprint, making it highly suitable for deployment in resource-constrained environments.

ShuﬄeNet [22] is an innovative convolutional neural network architecture designed to achieve high performance with significantly reduced computational cost. It introduces the concept of group convolutions and channel shuﬄing to efficiently utilize computational resources while maintaining strong representational power. By dividing the input channels into groups, ShuﬄeNet enables parallel processing of feature maps, reducing computational complexity. Additionally, channel shuﬄing operations are employed to enhance information exchange between groups, promoting feature diversity and improving network performance. ShuﬄeNet also utilizes depthwise separable convolutions and pointwise convolutions to further reduce the number of parameters and computation, making it highly efficient for deployment on resource-constrained devices. Through these design choices, ShuﬄeNet achieves competitive accuracy on image classification tasks while requiring fewer computational resources, making it well-suited for applications requiring lightweight and efficient deep neural networks.

SqueezeNet [23] is an efficient convolutional neural network architecture designed to balance high performance with minimal computational resources. Its key innovation lies in the “fire” module, which combines both 1 × 1 and 3 × 3 convolutions in a compact architecture to reduce the number of parameters while preserving representational capacity. By squeezing the input channels through 1 × 1 convolutions and then expanding them through both 1 × 1 and 3 × 3 convolutions, SqueezeNet achieves a significant reduction in model size without sacrificing accuracy. Additionally, SqueezeNet utilizes global average pooling and softmax activation. These design choices result in a highly efficient network architecture that is suitable for deployment on resource-constrained devices such as mobile phones and IoT devices.

Xception [23] is a convolutional neural network architecture introduced by Google, characterized by its depth-wise separable convolutions, which efficiently capture spatial and cross-channel dependencies while reducing computational complexity. It follows a similar design to Inception but replaces standard convolutions with depth-wise separable convolutions, which consist of a depth-wise convolution followed by a point-wise convolution. This separation allows the network to learn spatial features independently of channel-wise features, resulting in a more parameter-efficient architecture. Additionally, Xception employs skip connections to facilitate gradient flow and improve training stability, similar to residual networks. These design choices make Xception highly efficient while maintaining strong representational power.

### Performance evaluation metrics

The performance of the models was evaluated using a multitude of metrics that capture their true strengths and weaknesses. shows the Matthews correlation coefficient (MCC) when extended to more than two classes. Note that *t_j_* sums elements in row j of the confusion matrix, *p_j_* sums element in column j of the confusion matrix, c sums diagonal elements in the confusion matrix, *t^T^* is the transpose of vector *t*, and s = 758 (i.e., total number of sandfly images used in this work). The MCC is considered a more informative and reflective measure of performance in comparison to accuracy and F-score [24] as it includes all elements of the confusion matrix in its formula. However, the accuracy () and F-score () were included in the reported results for completeness. Furthermore, we reported the precision (i.e., the positive predictive value), specificity (i.e., true negative rate), and recall (i.e., sensitivity or true positive rate). Each of these metrics reveals different aspect of performance. For example, a 90% female *Ph. sergenti* precision means that out of all images returned by the model as female *Ph. sergenti*, 90% of the images are truly of that class and 10% are false positives. On the other hand, a 90% recall of the same class, means that 90% of the Sergenti female images were detected as such and 10% were detected as other species/gender (i.e. missed by the model).


$$\begin{array}{ccc} MCC=\frac{cs-t^{T}.P}{\sqrt{s^{2}-p^{T}p}\sqrt{s^{2}-t^{T}t}} & & \end{array}$$ (1)

where,


$$\begin{array}{cccc} C_{ij}\text{, is the confusion matrix element at row i and column j} & & & \\ t_{j}=\overset{6}{\underset{i=1}{\sum }}C_{ji}\;\text{, is the number of subjects truly belonging to class j } & & & \\ p_{j}=\overset{6}{\underset{i=1}{\sum }}C_{ij}\;\text{, is the number of times class j was predicted } & & & \\ c=\overset{6}{\underset{i=1}{\sum }}C_{ii}\;\text{, is the total number of correct predictions for all classes} & & & \\ s=\overset{6}{\underset{i=1}{\sum }}\overset{6}{\underset{j=1}{\sum }}C_{ij}\;\text{, is the total number of subjects from all classes} & & & \end{array}$$

$$\begin{array}{ccc} Accuracy=\frac{1}{6}\overset{6}{\underset{i=1}{\sum }}\frac{TP_{i}}{TP_{i}+TN_{i}+FP_{i}+FN_{i}} & & \end{array}$$ (2)

$$\begin{array}{ccc} \text{F-score = }\frac{1}{6}\overset{6}{\underset{i=1}{\sum }}\frac{2TP_{i}}{2TP_{i}++FP_{i}+FN_{i}} & & \end{array}$$ (3)

$$\begin{array}{ccc} Precision=\frac{1}{6}\overset{6}{\underset{i=1}{\sum }}\frac{TP_{i}}{TP_{i}+FP_{i}} & & \end{array}$$ (4)

$$\begin{array}{ccc} Recall=\frac{1}{6}\overset{6}{\underset{i=1}{\sum }}\frac{TP_{i}}{TP_{i}+FN_{i}} & & \end{array}$$ (5)

$$\begin{array}{ccc} Specificity=\frac{1}{6}\overset{6}{\underset{i=1}{\sum }}\frac{TN_{i}}{TN_{i}+FP_{i}} & & \end{array}$$ (6)

where,


$$\begin{array}{cccc} TP_{i}=C_{ii}\text{, is the number of correct predictions of class i} & & & \\ FP_{i}=\overset{6}{\underset{j=1}{\sum }}C_{ji}-C_{ii}\;\text{, is the number of false positives of class i } & & & \\ FN_{i}=\overset{6}{\underset{j=1}{\sum }}C_{ij}-C_{ii}\text{, is the number of false negatives of class i } & & & \\ TN_{i}=s-TP_{i}-FP_{i}-FN_{i}\;\text{, is the number of true negatives of class i} & & & \end{array}$$

### Performance evaluation setup

The twelve models were evaluated under the same conditions using the same software platform (i.e., Matlab R2023b). The same random seed was used for all algorithms. The separated pharynx and genital images were cropped and resized into a tensor of size 400 × 400 × 3. Fivefold cross-validation was used to counter any effect on performance resulting from the random split of the data. In this scheme, the data is separated into five equally-sized subsets (i.e., folds). Hence, each subset contains 20% of the dataset. Four folds are used for training and validation (i.e., 70% and 10%, respectively), and the fifth fold is used for testing as unseen data. This process is repeated so that each fold is used for testing the model that is trained and validated using the remaining four folds. The training and validation are done from scratch each time, so that the testing fold is truly considered unseen. The performance results represent the average and standard deviation from the five folds of testing.

**Table 1:** The six-way classification results for all CNN models using 5-fold cross-validation. The numbers represent the average of the 5 folds of testing  ±  standard deviation.

Model	Precision	Recall	Specificity	F-score	Accuracy	MCC
GoogLeNet	97.0% ( ± 1 . 9)	96.6% ( ± 1 . 7)	99.8% ( ± 0 . 4)	96.8% ( ± 1 . 9)	98.0% ( ± 1 . 2)	97.4% ( ± 1 . 5)
ResNet-18	96.6% ( ± 1 . 9)	96.2% ( ± 2 . 2)	99.6% ( ± 0 . 5)	96.0% ( ± 2 . 3)	97.6% ( ± 1 . 7)	96.8% ( ± 1 . 8)
ResNet-50	97.8% ( ± 1 . 6)	97.4% ( ± 1 . 8)	99.8% ( ± 0 . 4)	97.2% ( ± 2 . 0)	98.2% ( ± 1 . 1)	97.8% ( ± 1 . 3)
DarkNet-19	97.8% ( ± 1 . 6)	97.2% ( ± 1 . 6)	99.8% ( ± 0 . 4)	97.2% ( ± 2 . 0)	98.2% ( ± 1 . 1)	97.6% ( ± 1 . 1)
DarkNet-53	97.6% ( ± 1 . 9)	97.0% ( ± 2 . 0)	99.8% ( ± 0 . 4)	96.8% ( ± 2 . 2)	98.0% ( ± 1 . 4)	97.4% ( ± 1 . 5)
DenseNet	97.4% ( ± 1 . 5)	96.6% ( ± 1 . 7)	99.8% ( ± 0 . 4)	96.8% ( ± 1 . 6)	98.0% ( ± 1 . 2)	97.2% ( ± 1 . 3)
Inceptionv3	96.4% ( ± 1 . 7)	96.2% ( ± 1 . 5)	99.8% ( ± 0 . 4)	96.0% ( ± 1 . 6)	97.4% ( ± 1 . 5)	96.8% ( ± 1 . 6)
MobileNetv2	96.4% ( ± 2 . 1)	96.4% ( ± 2 . 4)	99.6% ( ± 0 . 5)	96.0% ( ± 2 . 5)	97.4% ( ± 1 . 5)	96.6% ( ± 1 . 7)
ShuﬄeNet	96.6% ( ± 2 . 4)	96.6% ( ± 2 . 3)	99.6% ( ± 0 . 5)	96.4% ( ± 2 . 3)	97.8% ( ± 1 . 3)	97.0% ( ± 1 . 4)
SqueezeNet	95.2% ( ± 0 . 8)	94.6% ( ± 1 . 5)	99.4% ( ± 0 . 5)	94.2% ( ± 1 . 5)	95.8% ( ± 1 . 3)	95.2% ( ± 1 . 8)
Xception	94.0% ( ± 2 . 0)	93.6% ( ± 1 . 5)	99.0% ( ± 0 . 0)	93.2% ( ± 2 . 6)	95.4% ( ± 1 . 1)	94.4% ( ± 1 . 7)
NasNetMobile	96.4% ( ± 2 . 3)	95.2% ( ± 1 . 9)	99.4% ( ± 0 . 5)	95.4% ( ± 2 . 3)	96.8% ( ± 1 . 8)	96.0% ( ± 1 . 9)

The network parameters were updated during training using the stochastic gradient descent with momentum (SGDM) algorithm, which was one of the options available and is known for its stability and fast convergence. No fine-tuning of the hyperparameters or the network solver was performed as the resulting performance was excellent. The minimum batch size was set to 16 as a compromise between training speed and required memory. The initial learning rate was 0.0003. The weight learn rate and the bias learn rate factors in the new learnable layer were set to 10. Thirty training epochs were found to be sufficient for stable training.

**Fig 4:**
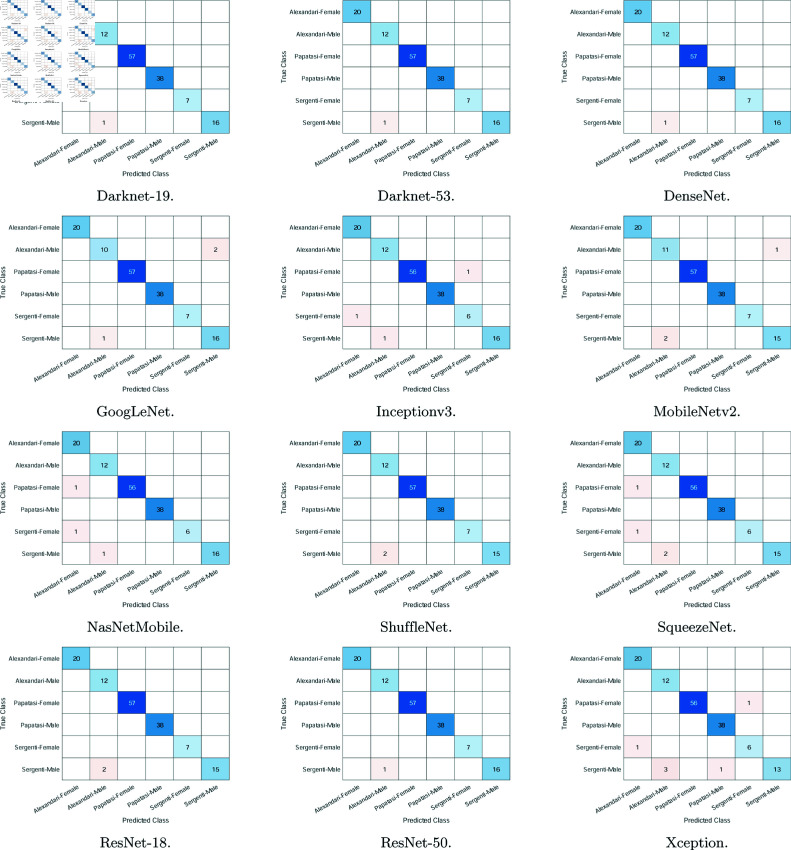
The confusion matrices using the 12 deep learning models. The matrices show the results from the last fold of testing.

## Results and discussion

Table 1 shows the results for all CNN models using fivefold cross-validation. All models achieved an MCC value greater than 95%, except for Xception, which performed closely at 94.4% MCC. All of the other performance metrics followed suit. Specificity was very high (i.e.,  > 99.0%), which indicates there is an extremely low number of false positives across all species and sexes. Although the average results are reported, it should be noted that for all indices, no value in the table dropped below 90% for any run (i.e., fold) across all algorithms.

These results are further corroborated by the confusion matrices in Fig 4. In these matrices, the rows represent the true class and the columns represent the predicted class. Hence, diagonal elements represent the number of correct predictions. In most cases, two classes or fewer witnessed mis-predictions (i.e., either false positives or false negatives), and these are most common with a *Ph. sergenti* male image being misclassified as an *Ph. alexandri* male. All methods witnessed this error with three or fewer images. Furthermore, images of the *Ph. alexandri* female, papatasi female/male, and *Ph. sergenti* female classes were almost always correctly classified. In addition, the *Ph. alexandri* class, especially the male one, received the highest number of false positive predictions (i.e., other images classified as *Ph. alexandri*). Nonetheless, the number of errors is small in comparison to the total number of test images, and the matrices do confirm the excellent results reported in Table 1.

**Table 2:** The species classification results (i.e., 3-way classification) for all CNN models using 5-fold cross-validation. The numbers represent the average of the 5 folds of testing  ±  standard deviation.

Model	Precision	Recall	Specificity	F-score	Accuracy	MCC
GoogLeNet	97.2% ( ± 1 . 9)	97.0% ( ± 2.2)	99.0% ( ± 0 . 7)	97.0% ( ± 2)	98.2% ( ± 1 . 3)	96.8% ( ± 2 . 3)
ResNet-18	96.0% ( ± 1 . 2)	96.2% ( ± 1 . 1)	99.0% ( ± 0 . 0)	95.8% ( ± 1 . 3)	97.4% ( ± 0 . 5)	95.8% ( ± 1 . 3)
ResNet-50	97.6% ( ± 1 . 7)	97.6% ( ± 1 . 8)	99.2% ( ± 0 . 4)	97.4% ( ± 1 . 9)	98.6% ( ± 1 . 1)	97.4% ( ± 1 . 9)
DarkNet-19	97.2% ( ± 0 . 8)	97.2% ( ± 1 . 6)	99.0% ( ± 0 . 0)	97.2% ( ± 1 . 3)	98.4% ( ± 0 . 9)	97.0% ( ± 1 . 4)
DarkNet-53	97.6% ( ± 1 . 1)	97.0% ( ± 1 . 2)	99.0% ( ± 0 . 0)	97.0% ( ± 1 . 2)	98.2% ( ± 0 . 8)	97.0% ( ± 1 . 2)
DenseNet	97.0% ( ± 1 . 7)	96.6% ( ± 2 . 2)	98.8% ( ± 0 . 4)	97.0% ( ± 1 . 7)	98.2% ( ± 1 . 3)	96.6% ( ± 2 . 2)
Inceptionv3	96.2% ( ± 1 . 5)	96.0% ( ± 1 . 5)	99.2% ( ± 0 . 4)	96.0% ( ± 1 . 4)	97.2% ( ± 1 . 7)	96.6% ( ± 1 . 6)
MobileNetv2	96.2% ( ± 1 . 5)	96.0% ( ± 2 . 1)	98.8% ( ± 0 . 4)	95.8% ( ± 1 . 8)	97.4% ( ± 1 . 1)	95.8% ( ± 1 . 8)
ShuﬄeNet	96.8% ( ± 1 . 3)	97.0% ( ± 1 . 7)	99.0% ( ± 0 . 0)	96.6% ( ± 1 . 7)	98.0% ( ± 1 . 0)	96.6% ( ± 1 . 7)
SqueezeNet	97.0% ( ± 1 . 4)	96.6% ( ± 1 . 5)	98.8% ( ± 0 . 4)	96.6% ( ± 1 . 5)	97.8% ( ± 1 . 3)	96.2% ( ± 2 . 2)
Xception	94.0% ( ± 2 . 0)	93.4% ( ± 1 . 5)	99.0% ( ± 0 . 0)	93.4% ( ± 2 . 6)	95.0% ( ± 1 . 1)	94.4% ( ± 1 . 7)
NasNetMobile	96.4% ( ± 2 . 1)	95.0% ( ± 1 . 9)	99.0% ( ± 0 . 4)	95.2% ( ± 2 . 4)	96.4% ( ± 1 . 7)	96.0% ( ± 1 . 5)

**Fig 5:**
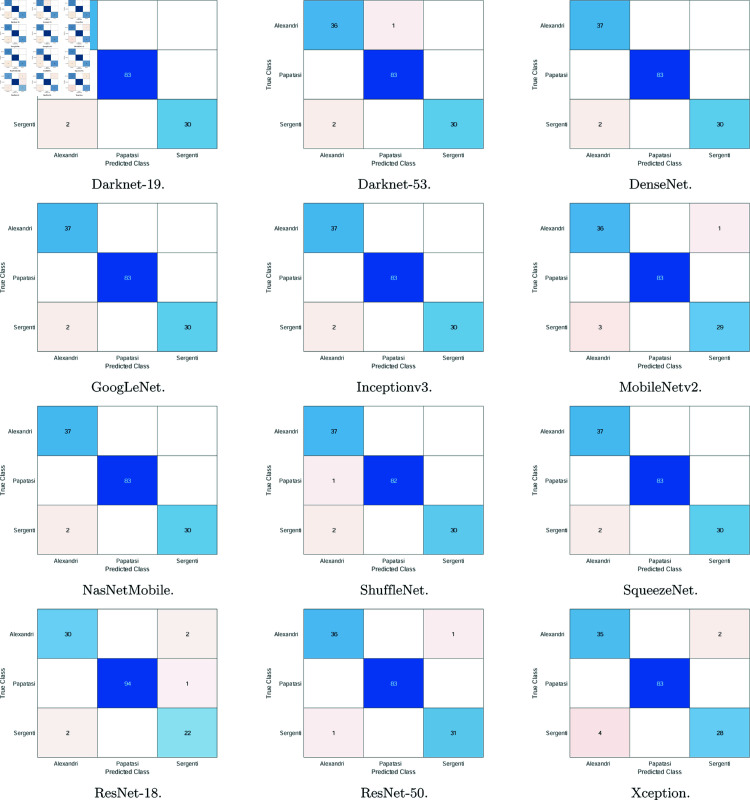
The confusion matrices using the 12 deep learning models for specie only classification (i.e., three-way). The matrices show the results from the last fold of testing.

Two further experimental combinations were evaluated. First, all images were clustered into male or female classes regardless of species (i.e., two classes only instead of six). Such division resulted in perfect classification by all models (i.e., 100% values across all models). This was to be expected as the male/female differences are apparent and easy to discern by the models or any human operator. Second, the images were grouped based on species alone (i.e., three classes corresponding to *Ph. papatasi*, *Ph. sergenti*, and *Ph. alexandri*). Table 2 shows the three-way classification results, which demonstrate great ability to determine the specific species. The results are further confirmed by the confusion matrices in Fig 5 with very few errors.

AI for species identification faces key limitations, primarily due to constraints in training data and model generalization. Limited datasets for rare or understudied species can lead to overfitting, reducing accuracy and generalizability [25]. Additionally, AI models may struggle with geographic diversity, where species appearances vary across regions, thus impacting reliability [26]. Distinguishing between cryptic or similar-looking species remains challenging without specialized data, often resulting in misidentifications. Bias in datasets, where certain species are overrepresented, further skews model predictions [27]. The computational demands of deep learning models add practical barriers, limiting their use in resource-constrained settings [28], although this concern is being ironed out by advances in commercially available computational power. Environmental variability, such as changes in lighting and background, also affects model robustness in field conditions, posing additional challenges [27]. Moreover, citizen science projects and collaborative platforms, such as iNaturalist and eBird, have greatly expanded the availability of labeled images, enabling the creation of larger, more diverse datasets that support AI training and validation [29]. Recently, few-shot learning has emerged as a valuable tool for identifying species with very limited data, as it allows models to learn distinguishing features from just a few examples, which is especially useful for rare species [30]. In addition, incorporation of metadata and contextual information (e.g., location, season) into AI models has been shown to enhance identification by providing additional clues beyond visual features alone [27].

In this work, we have demonstrated that it is possible to develop highly accurate deep learning AI models for the taxonomy and gender identification of sandfly species. The extensive evaluation across multiple models and performance metrics reveals the robustness of this method for the task at hand, indicating great potential to streamline this process for real-life applications. Future work in this domain will aim to enhance the dataset and the resulting models to include more species and more images per species. Furthermore, integrating these models into a standalone application or microscopic imaging applications would provide a complete hassle-free service to entomologists. Moreover, the methods in this work can be readily extended to the taxonomy of other insects (e.g., mosquitoes), given the availability of relevant data. In addition, a more powerful application feature that does not rely on the user to crop or stitch the images would be desirable, but this is the subject of image segmentation and object detection algorithms.

## Conclusion

Artificial intelligence is increasingly applied across various fields, yet entomology still holds significant potential for leveraging its benefits. In this study, we developed a deep learning system capable of accurately determining the gender and species of three sandfly genera (*Ph. alexandri*, *Ph. papatasi*, and *Ph. sergenti*). Using convolutional neural networks, transfer learning, and early fusion of genital and pharynx images, our system achieved classification accuracy above 95% across multiple metrics. This high accuracy highlights the potential of our approach to improve disease surveillance and control, manage sandfly populations, and support further research and epidemiological studies.

Our results demonstrate that deep learning techniques can be effectively applied to the field of medical entomology, providing an automated and reliable method for sandfly identification. This advancement is crucial for vector-borne disease research and control efforts, as accurate identification of sandfly species and gender is essential for understanding disease dynamics and implementing targeted interventions.

Moreover, our study establishes a robust framework that can be adapted and extended to other vector species, offering a scalable solution for broader applications in the control and prevention of vector-borne diseases. Future work could explore the integration of additional morphological features and the application of this framework to other vectors of medical importance, further expanding the impact and utility of deep learning in entomological research and public health.

## References

[1] Singh NS, Phillips-Singh D. Pharyngeal armature, an important morphometric tool for the taxonomic studies of Phlebotominae sandflies (Phlebotomidae: Diptera). J Entomol Res 2022;46(1):206–11. doi: 10.5958/0974-4576.2022.00037.8

[2] Maroli M, Feliciangeli MD, Bichaud L, Charrel RN, Gradoni L. Phlebotomine sandflies and the spreading of leishmaniases and other diseases of public health concern. Med Vet Entomol 2013;27(2):123–47. doi: 10.1111/j.1365-2915.2012.01034.x 22924419

[3] Ready PD. Biology of phlebotomine sand flies as vectors of disease agents. Annu Rev Entomol 2013;58(1):227–50. doi: 10.1146/annurev-ento-120811-153557 23317043

[4] Seccombe A, Ready P, Huddleston L. A catalogue of the old world phlebotomine sandflies (Diptera: Psychodidae, Phlebotominae). 1993.

[5] Akhoundi M, Kuhls K, Cannet A, Votýpka J, Marty P, Delaunay P, et al. A historical overview of the classification, evolution, and dispersion of leishmania parasites and sandflies. PLoS Negl Trop Dis 2016;10(3):e0004349. doi: 10.1371/journal.pntd.0004349 26937644 PMC4777430

[6] Halada P, Hlavackova K, Dvorak V, Volf P. Identification of immature stages of phlebotomine sand flies using MALDI-TOF MS and mapping of mass spectra during sand fly life cycle. Insect Biochem Mol Biol. 2018;93:47–56. doi: 10.1016/j.ibmb.2017.12.005 29248738

[7] Mathis A, Depaquit J, Dvořák V, Tuten H, Bañuls A-L, Halada P, et al. Identification of phlebotomine sand flies using one MALDI-TOF MS reference database and two mass spectrometer systems. Parasit Vectors. 2015;8:266. doi: 10.1186/s13071-015-0878-2 25957576 PMC4432514

[8] Kavur H. TRsandflies: A web-based software for the morphometric identification of sand flies in turkey. J Med Entomol 2021;58(3):1149–56. doi: 10.1093/jme/tjaa275 33331881

[9] Fraiwan M, Al-Kofahi N, Ibnian A, Hanatleh O. Detection of developmental dysplasia of the hip in X-ray images using deep transfer learning. BMC Med Inform Decis Mak 2022;22(1):216. doi: 10.1186/s12911-022-01957-9 35964072 PMC9375244

[10] Fraiwan MA, Abutarbush SM. Using artificial intelligence to predict survivability likelihood and need for surgery in horses presented with acute abdomen (Colic). J Equine Vet Sci. 2020;90:102973. doi: 10.1016/j.jevs.2020.102973 32534764

[11] Fraiwan M, Faouri E, Khasawneh N. Classification of corn diseases from leaf images using deep transfer learning. Plants (Basel) 2022;11(20):2668. doi: 10.3390/plants11202668 36297692 PMC9609100

[12] Hartbauer M. Artificial neuronal networks are revolutionizing entomological research. J Appl Entomol 2024;148(2):232–51. doi: 10.1111/jen.13227

[13] Cannet A, Simon-Chane C, Histace A, Akhoundi M, Romain O, Souchaud M, et al. Species identification of phlebotomine sandflies using deep learning and wing interferential pattern (WIP). Sci Rep 2023;13(1):21389. doi: 10.1038/s41598-023-48685-2 38049590 PMC10696019

[14] Carranza-Rojas J, Goeau H, Bonnet P, Mata-Montero E, Joly A. Going deeper in the automated identification of Herbarium specimens. BMC Evol Biol 2017;17(1):181. doi: 10.1186/s12862-017-1014-z 28797242 PMC5553807

[15] Deng J, Dong W, Socher R, Li L, Li K, Fei-Fei L. ImageNet: A large-scale hierarchical image database. In: 2009 IEEE conference on computer vision and pattern recognition. 2009. p. 248–255.

[16] Redmon J. Darknet: Open source neural networks in C. 2013–2016. Available from: http://pjreddie.com/darknet/ [cited 2024 January 30].

[17] Huang G, Liu Z, Van Der Maaten L, Weinberger KQ. Densely connected convolutional networks. In: 2017 IEEE conference on computer vision and pattern recognition (CVPR). 2017. p. 2261–2269.

[18] Szegedy C, Liu W, Jia Y, Sermanet P, Reed S, Anguelov D. Going deeper with convolutions. In: 2015 IEEE conference on computer vision and pattern recognition (CVPR). 2015. p. 1–9.

[19] Szegedy C, Ioffe S, Vanhoucke V, Alemi AA. Inception-v4, Inception-ResNet and the impact of residual connections on learning. In: Proceedings of the thirty-first AAAI conference on artificial intelligence. AAAI’17. AAAI Press; 2017. p. 4278–4284.

[20] Sandler M, Howard A, Zhu M, Zhmoginov A, Chen L. MobileNetV2: Inverted residuals and linear bottlenecks. In: 2018 IEEE/CVF conference on computer vision and pattern recognition. 2018. p. 4510–4520.

[21] Zoph B, Vasudevan V, Shlens J, Le QV. Learning transferable architectures for scalable image recognition. In: 2018 IEEE/CVF conference on computer vision and pattern recognition. 2018. p. 8697–8710. doi: 10.1109/cvpr.2018.00907

[22] Zhang X, Zhou X, Lin M, Sun J. ShuﬄeNet: An extremely efficient convolutional neural network for mobile devices. In: 2018 IEEE/CVF conference on computer vision and pattern recognition. 2018. p. 6848–6856.

[23] Iandola FN, Moskewicz MW, Ashraf K, Han S, Dally WJ, Keutzer K. SqueezeNet: AlexNet-level accuracy with 50x fewer parameters and <1MB model size. arXiv. 2016.

[24] Stoica P, Babu P. Pearson–Matthews correlation coefficients for binary and multinary classification. Signal Process. 2024;222:109511. doi: 10.1016/j.sigpro.2024.109511

[25] Wäldchen J, Mäder P. Plant species identification using computer vision techniques: a systematic literature review. Arch Comput Methods Eng 2018;25(2):507–43. doi: 10.1007/s11831-016-9206-z 29962832 PMC6003396

[26] Mac Aodha O, Gibb R, Barlow KE, Browning E, Firman M, Freeman R, et al. Bat detective-Deep learning tools for bat acoustic signal detection. PLoS Comput Biol 2018;14(3):e1005995. doi: 10.1371/journal.pcbi.1005995 29518076 PMC5843167

[27] Gomez Villa A, Salazar A, Vargas F. Towards automatic wild animal monitoring: Identification of animal species in camera-trap images using very deep convolutional neural networks. Ecol Inform. 2017;32–32. doi: 10.1016/j.ecoinf.2017.07.004

[28] Schmidhuber J. Deep learning in neural networks: an overview. Neural Netw. 2015;61:85–117. doi: 10.1016/j.neunet.2014.09.003 25462637

[29] Van Horn G, Mac Aodha O, Song Y, Cui Y, Sun C, Shepard A, et al. The iNaturalist species classification and detection dataset. In: 2018 IEEE/CVF conference on computer vision and pattern recognition. 2018. doi: 10.1109/cvpr.2018.00914

[30] Sung F, Yang Y, Zhang L, Xiang T, Torr PHS, Hospedales TM. Learning to compare: relation network for few-shot learning. In: 2018 IEEE/CVF conference on computer vision and pattern recognition. 2018. doi: 10.1109/cvpr.2018.00131

